# Finding Sustainable Deep Eutectic Solvents for an Efficient Separation of CO_2_ and NH_3_ in Melamine Production with Soft-SAFT

**DOI:** 10.1021/acssuschemeng.5c06167

**Published:** 2025-09-04

**Authors:** Luan Vittor Tavares Duarte de Alencar, Sabrina Belén Rodríguez-Reartes, Frederico Wanderley Tavares, Fèlix Llovell

**Affiliations:** † Department of Chemical Engineering, ETSEQ, 16777 Universitat Rovira i Virgili, Avinguda Països Catalans 26, 43007 Tarragona, Spain; ‡ Programa de Engenharia Química (PEQ/COPPE), Universidade Federal do Rio de Janeiro (UFRJ), Athos da Silveira Ramos Avenue, 149-Block G, Ilha do Fundão, 21941-909 Rio de Janeiro, RJ, Brazil; § Departamento de Ingeniería Química, Universidad Nacional del Sur (UNS), Avda. Alem 1253, Bahía Blanca (8000), Argentina; ∥ Planta Piloto de Ingeniería Química—PLAPIQUI (UNS-CONICET), Camino “La Carrindanga” km 7, Bahía Blanca 8000, Argentina; ⊥ Engenharia de Processos Químicos e Bioquímicos, Escola de Química (EPQB), Universidade Federal do Rio de Janeiro (UFRJ), Athos da Silveira Ramos Avenue, 149-Block E, Ilha do Fundão, 21941-909 Rio de Janeiro, RJ, Brazil

**Keywords:** deep eutectic solvents, solubility, soft-SAFT, carbon dioxide (CO_2_), ammonia (NH_3_), gas separation

## Abstract

Industrial processes frequently release tail gases containing harmful components, such as carbon dioxide (CO_2_) and ammonia (NH_3_), with significant environmental implications. Effective separation and recovery of these gases are critical for reducing pollution and enhancing resource efficiency. This paper investigates the use of deep eutectic solvents (DESs) to capture and separate CO_2_ and NH_3_ from melamine production tail gases, offering an alternative to traditional methods such as coproduction systems and water scrubbing. Choline chloride ([Ch]­Cl)-based solvents with ethylene glycol (EG), urea (UR), and glycerol (GL) are assessed for selective gas absorption using the soft-SAFT Equation of State (EoS). This approach has been used to model the solubility of CO_2_ and NH_3_ in these DESs and evaluate key properties, including absorption isotherms, enthalpy, entropy of dissolution, effective Henry’s constants, and ideal selectivity. Results reveal that the presence of EG enhances the NH_3_ absorption capacity, while UR shows higher CO_2_ absorption. From this information, the competitive selectivity in a standard melamine tail gas mixture (60% NH_3_ and 40% CO_2_ in mole fraction) and DESs has been predicted. Among them, [Ch]­Cl:EG shows the best selectivity, and the 1:7 proportion provides the optimal value for NH_3_/CO_2_ separation. This work highlights the potential of DESs for efficient gas separation, emphasizing the utility of molecular modeling as a precursor to experimental validation in the design of sustainable separation processes.

## Graphical abstract

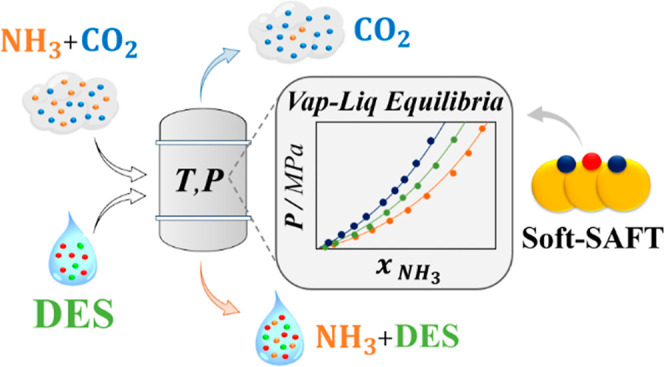

## 1 Introduction

Melamine (C_3_H_6_N_6_) is a vital industrial raw material extensively utilized in manufacturing thermal insulation compounds, adhesives, and fibers.^1^ Typically, the production of one ton of melamine through the decomposition and condensation of urea results in approximately 2.2 tons of tail gas, characterized by high concentrations of both NH_3_ and CO_2_.^2^ Annually, the global production of melamine exceeds 1.59 million tons, leading to substantial exhaust greenhouse gas emissions.^3^ This not only contributes to global warming, but also wastes valuable resources, as NH_3_ is an important chemical building block used as a versatile agent in preparing many fine chemicals.^4,5^ Therefore, an efficient separation and recovery of these gases can mitigate environmental impacts, enhance resource efficiency, and contribute to sustainable industrial practices.

According to literature reports, two primary methods have been developed for the simultaneous separation of NH_3_ and CO_2_ from gas mixtures. The first method involves coproducing urea or ammonium bicarbonate.^6,7^ However, maintaining material balance in the coproduction system is challenging, and the excess of ammonia cannot be effectively utilized.^3^ The second method is water scrubbing, which is effective in separating NH_3_ and CO_2_, but involves high utility consumption, making it an energy-intensive and costly process.^8^

A practical alternative method for NH_3_ recovery from tail gas streams in melamine production involves absorption into ionic liquids (ILs). In particular, imidazolium-based ILs have proven to be cost-efficient compared to traditional water scrubbing techniques.^8,9^ Additionally, the viability of IL-based NH_3_ recovery from melamine tail gas has been demonstrated through the construction of the first industrial test pilot plant worldwide, developed by the Institute of Process Engineering, Chinese Academy of Sciences.^2^ This plant, with a 50 N m^3^ h^–1^ capacity and 3500 h of operation,^8^ uses a specially designed IL with multiple hydrogen bonding sites, selected from a series of hydroxyl, protic, and metal ILs, to optimize NH_3_ selectivity, stability, and energy efficiency.

The successful application of ILs comes at a certain cost associated with the synthesis of the solvent and some environmental concerns. Deep Eutectic Solvents (DESs) offer a promising alternative for gas capture, with lower toxicity, corrosivity and cost.^10^ A DES consists of a liquid mixture of two or more hydrogen bonding components, which exhibits a significant depression in its melting point compared to that of their constituents, due to the hydrogen bond interactions between the hydrogen bond donor (HBD) and the hydrogen bond acceptor (HBA) compounds conforming the system.^11,12^ The unique properties of DESs have garnered significant attention in various fields, including separation processes and gas capture technologies.^13–19^ These solvents enable selective extraction, offering a sustainable alternative to traditional solvents. Extensive literature supports the adoption of DESs for capturing CO_2_ ^15,20–23^ and NH_3_.^24–27^

The capability of DESs for gas absorption will depend on the selection of the two compounds forming the eutectic mixture and the proportion between them. Given the many possibilities, reliable predictive models are needed to quickly screen key thermodynamic properties, such as gas solubility and selectivity between gases. Many different thermodynamic models are available, ranging from quantum and molecular simulations, until cubic and molecular-based equations of state (EoS). Among them, SAFT-type equations of state^28,29^ are attractive for modeling DESs due to their ability to explicitly account for hydrogen bonding. For instance, the well-known PC-SAFT version^30^ has been successfully applied to predict CO_2_ solubility in various DESs.^31,32^ Also, the soft-SAFT EoS,^33^ has also been used to describe the absorption of gases in DESs, showing accurate predictions for the solubilities of CO_2_ ^34–36^ and fluorinated gases,^37,38^ among other modeled compounds.

This work aims to analyze the solubility of CO_2_ and NH_3_ in three different choline chloride ([Ch]­Cl)-based DESs formed with ethylene glycol (EG), urea (UR), and glycerol (GL). The ideal selectivity of NH_3_ over CO_2_ in these DESs is examined, along with the prediction of absorption isotherms, the enthalpy and entropy of dissolution, and the Henry’s constants. Furthermore, the study predicts the competitive selectivity of NH_3_ over CO_2_ in multicomponent mixtures based on melamine tail gas and DESs. From these analyzes, a rational discussion is made to identify the most suitable DES for efficient NH_3_ recovery from the tail gas, supporting the initial phase of design of an industrial gas separation unit.

## 2 Methodology

### 2.1 The Soft-SAFT Equation of State

The general expression for the soft-SAFT EoS^33^ computes the residual Helmholtz energy (*A* ^res^) by summing different microscopic contributions to a given molecule, as

1 $$A^{res}=A^{ref}+A^{chain}+A^{assoc}+A^{polar}$$

In eq 1, the reference term (*A* ^ref^) accounts for segment–segment interactions via the Lennard-Jones potential, calculated using the Johnson et al. EoS^39^ within soft-SAFT. The chain term (*A* ^chain^) describes the connectivity of segments into chains, while the association term (*A* ^assoc^) captures strong, short-range directional interactions from hydrogen bonding. Both terms stem from Wertheim’s first-order thermodynamic perturbation theory^40–43^ and are formally identical across all SAFT variants. An additional polar term (*A* ^polar^) is included to capture long–range interactions such as CO_2_ quadrupolar forces, based on the theory of Gubbins and Twu^44^ (initially developed for spherical molecules) and later extended to chain fluids following the ideas of Jog et al.^45^ Further details about the model and the inclusion of the polar term can be found in the original contributions.^46,47^

Thermodynamic modeling with soft-SAFT requires molecular parameters describing structural and energetic characteristics. For nonassociating compounds, three parameters are used: chain length (*m*), segment diameter (σ), and dispersive energy (ε). For associating systems, two extra parameters, the association volume (κ^HB^) and energy (ε^HB^), are included. These are typically fitted to experimental data. For polar compounds, the polar moment (μ/*Q*) and the polar segment fraction (*x*_p_) are specified. The polar moment is often taken from experimental sources while *x*_p_ is set a priori based on physical arguments.^33,46^

The extension to mixtures is straightforward for the chain, association, and polar terms, as they are specifically written for a multicomponent case. For the reference term, the van der Waals one-fluid theory is applied, using unlike size and energy parameters of the Lennard-Jones fluid obtained via the generalized Lorentz–Berthelot rules (eqs 2 and 3)­

2 $$\sigma_{ij}=\eta_{ij}\frac{\left(\sigma_{ii}+\sigma_{jj}\right)}{2}$$

3 $$\varepsilon_{ij}=\xi_{ij}\sqrt{\varepsilon_{ii}\varepsilon_{jj}}$$

where η_*ij*_ and ξ_*ij*_ are the adjustable size and energy binary interaction parameters between species *i* and *j*. These parameters account for asymmetry and nonidealities between the different natures of the mixture compounds. They can be fitted to binary experimental data if predictions from the pure components (η_*ij*_ = ξ_*ij*_ = 1) are unsatisfactory.

For mixtures of compounds with hydrogen-bonding interactions, cross-association between different molecules or functional groups within the same type of molecules are calculated using combining rules, in a similar manner as done in eqs 2 and 3. When energies and volumes between a site type A in component *i* and a site type B in component *j* are required, the following equations are applied

4 $$\varepsilon_{AB,ij}^{HB}=\alpha_{ij}^{HB}\sqrt{\varepsilon_{AB,ii}^{HB}\varepsilon_{AB,jj}^{HB}}$$

5 $$k_{AB,ij}^{HB}={\left(\frac{\sqrt[{3}]{k_{AB,ii}^{HB}}+\sqrt[{3}]{k_{AB,jj}^{HB}}}{2}\right)}^{3}$$

where α_*ij*_^HB^ acts as a correction factor for the cross-association energy and can be fitted to experimental binary data if the default assumption (α_*ij*_^HB^ = 1) does not provide a suitable description of the system.

### 2.2 Molecular Models

To accurately describe the thermodynamic behavior of the studied systems, each compound was represented using a molecular model tailored to its structural and interaction characteristics. A summary of these representations, including molecular structures and corresponding soft-SAFT models used in this work are summarized in Figure S1 of the Supporting Information. Following a previous contribution,^48^ [Ch]­Cl:UR (1:2), [Ch]­Cl:EG (1:2), and [Ch]­Cl:GL (1:2) were modeled using the individual-component approach, where each entity is characterized by a specific set of parameters. All compounds were modeled with two different association sites (A and B), to simulate the positively and negatively charged regions in the molecule, respectively. A-B interactions are allowed to represent the HBA–HBD associations. In prior works, the parameters of EG^49^ and GL^50^ had already been adjusted to vapor–liquid equilibrium (VLE) data (saturated liquid density and vapor pressure), while the parameters for [Ch]Cl and UR were later obtained by fitting to DES single-phase density data,^48^ as these fluids exhibit negligible vapor pressures. These parameters were used in this current work.

Additionally, CO_2_ is modeled as a Lennard-Jones (LJ) nonassociating chain, but explicitly incorporates quadrupolar interactions through the polar term. For CO_2_, *x*_p_ was fixed at 1/3, representing that only one-third of the molecule is affected by the quadrupolar interactions. Departing from the experimental value, the effective quadrupole moment for CO_2_ (*Q* = 4.40 × 10^–40^ C·m^–2^) was refined from fitting. The final set of molecular parameters for CO_2_ was taken from Dias et al.^51^ Finally, a four-site model is proposed for NH_3_, following the ideas of Llovell et al.,^52^ with three H-type sites representing the hydrogen atoms and one e-type site representing the lone pair of electrons on nitrogen. Only e–H associating interactions are allowed. Although a set of soft-SAFT parameters is available in the literature for NH_3_,^52^ some deviations in the description of the saturated liquid density were found at the range of temperatures of interest for this work (280–360 K), with an % AAD of 7.03%. This temperature range is crucial for the current study on the absorption of NH_3_ from tail gas in melamine production with DESs. Consequently, a new parametrization has been carried out for NH_3_ using saturated liquid density and vapor pressure data, achieving a better fit to the VLE of ammonia in the range of interest, with an % AAD of 1.02% in the 275–375 K range. The description of the VLE equilibrium properties of ammonia with both parameter sets can be found in Figure S2 of the Supporting Information (see also Table S1 for the corresponding parameter values). A summary of the parameters for all compounds is provided in Table 1.

**Table 1:** Soft-SAFT Molecular Parameters Optimized for the Species That Form the Deep Eutectic Solvents and the Gases Studied in This Work

compound	*M*_w_ (g/mol)	*m*	σ (Å)	ε/*k*_B_ (K)	ε^HB^/*k*_B_ (K)	*k* ^HB^ (Å^3^)	No. of sites	Reference
[Ch]Cl	139.63	5.096	3.401	428.40	3384	2100	1 + 1	^48^
UR	60.06	2.458	3.090	420.70	3384	2100	1 + 1	^48^
EG	62.07	1.751	3.668	326.05	4384	4195	1 + 1	^49^
GL^a^	92.09	2.397	3.638	392.95	4945	2250	1 + 1	^50^
CO_2_	44.01	1.571	3.184	160.2				^51^
NH_3_	17.04	1.873	2.679	236.5	1136	1498	3 + 1	this work

a Additional molecular parameters for the polar contribution: *Q* = 4.40 × 10^–40^ C·m^–2^, *x*_p_ = 1/3.

## 3 Results

### 3.1 Solubility of CO_2_ in [Ch]Cl Based-DESs

A quantitative and reliable description of the process requires accounting for the binary interactions between all components in the system. As DESs are intrinsically binary mixtures (although it is possible to have also ternary or multicomponent DESs), it is necessary to consider the interactions of each gas with both constituents. Consequently, and before proceeding to evaluate the solubility of CO_2_ in the selected DESs, the binary system CO_2_-EG, for which experimental data were available,^53^ has been first examined.

Results concerning the soft-SAFT description of the VLE for the mixture between EG and CO_2_ at different temperatures are depicted in Figure 1a and compared to experimental data.^53^ As can be seen, an excellent agreement is achieved (%AAD of 3.06%) by setting a constant binary parameter $\xi_{EG-{CO}_{2}}$ = 0.886. This parameter was fitted to data at 298.15 K and used to predict the other three isotherms at 288.15, 308.15, and 318.15 K, ensuring that a temperature-independent parameter can accurately reproduce the system at different conditions.

**Figure 1:**
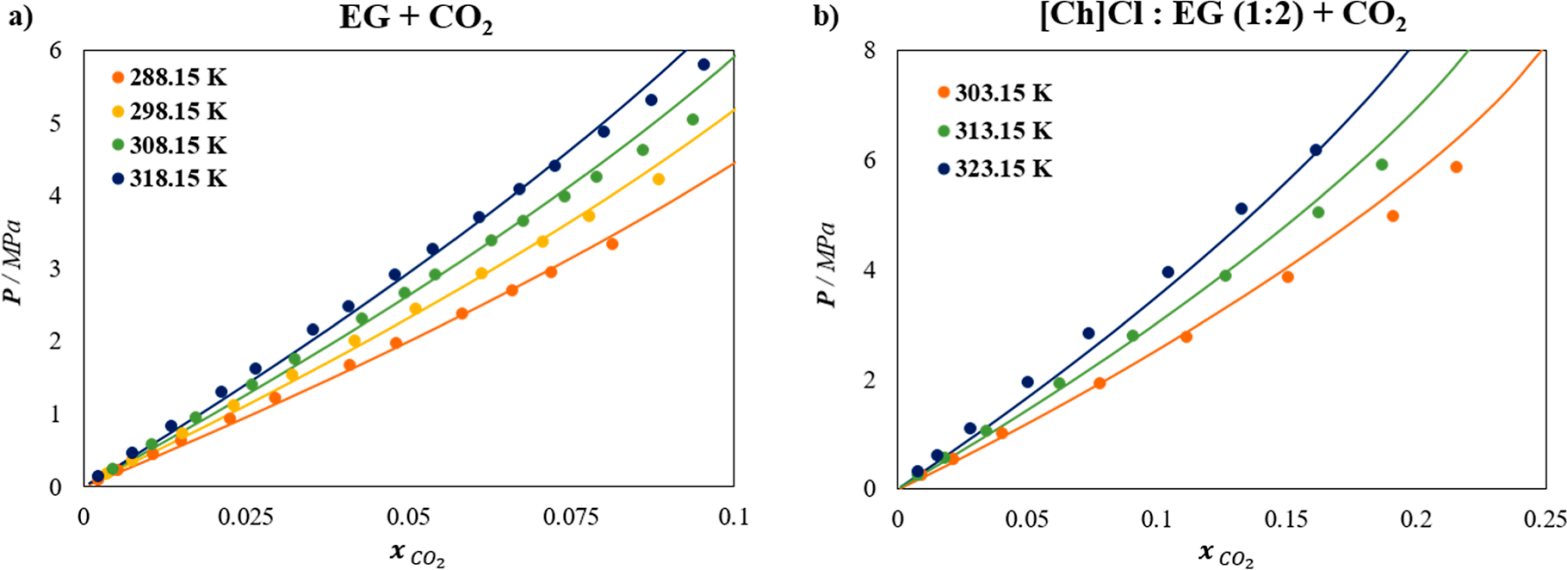
Solubility of CO_2_ at different temperatures in (a) EG and (b) [Ch]­Cl:EG (1:2) DES. Symbols represent experimental data^53,54^ and lines are soft-SAFT calculations.

The next step concerns the description of the CO_2_ solubility in the selected DES [Ch]­Cl:EG 1:2, considering it as a ternary mixture. HBA–HBD interactions are always described without involving any binary parameter (ξ_HBA–HBD_ = 1.00), regardless of the type of HBD employed. Moreover, the $\xi_{EG-{CO}_{2}}$ value previously fitted, accounting for the HBD–CO_2_ interaction, has been transferred to address the EG–CO_2_ interaction in the ternary mixture DES + CO_2_, while the ξ parameter describing the interaction between [Ch]Cl (HBA) and CO_2_, has been fitted to the ternary data. In particular, the intermediate temperature isotherm was used to obtain an optimum $\xi_{\left[Ch\right]Cl-{CO}_{2}}$ value of 1.10, while the other two isotherms were predicted using this value. The results are plotted in Figure 1b, evidencing excellent agreement with the CO_2_ solubility data across all isotherms, with an % AAD of 6.02%.

The subsequent step involves describing the CO_2_ solubility in other DESs with different HBDs (GL and UR). The $\xi_{\left[Ch\right]Cl-{CO}_{2}}$ previously fitted (see Figure 1b) has been transferred to the other two [Ch]­Cl-based DESs, provided that this binary interaction is independent of the HBD. Additionally, the $\xi_{HBD-{CO}_{2}}$ values, corresponding to UR and GL with CO_2_, have been fitted to the corresponding ternary data. In both cases, the intermediate temperature isotherm was used to obtain optimal ξ values, while the other two isotherms were predicted. A summary of the necessary ξ values to quantitatively describe the CO_2_ solubility in DESs is provided in Table S2 of the Supporting Information.

No further adjustments are necessary for the Lorentz combining rule (η = 1), which is consistently applied across all binary combinations, except for [Ch]­Cl:UR (1:2), where a constant η value of 0.9 between UR and CO_2_ is necessary. This variation may potentially be attributed to the findings from Crespo et al.,^50^ which suggested that different types of hydrogen bonds may form in the [Ch]­Cl:UR (1:2) mixture beyond what simplified models can accurately capture. In our approach, these interactions have been simplified to two association sites each for [Ch]Cl and UR to facilitate the transferability of soft-SAFT parameters, possibly underestimating specific interactions. Figure 2 displays the results for the [Ch]­Cl-based DESs with UR, and GL in a (1:2) ratio. Overall, this approach yields an excellent description of the CO_2_ solubility across various isotherms for the DESs, with an % AAD of 3.24% and 5.17% for [Ch]­Cl:GL (1:2) and [Ch]­Cl:UR (1:2), respectively.

**Figure 2:**
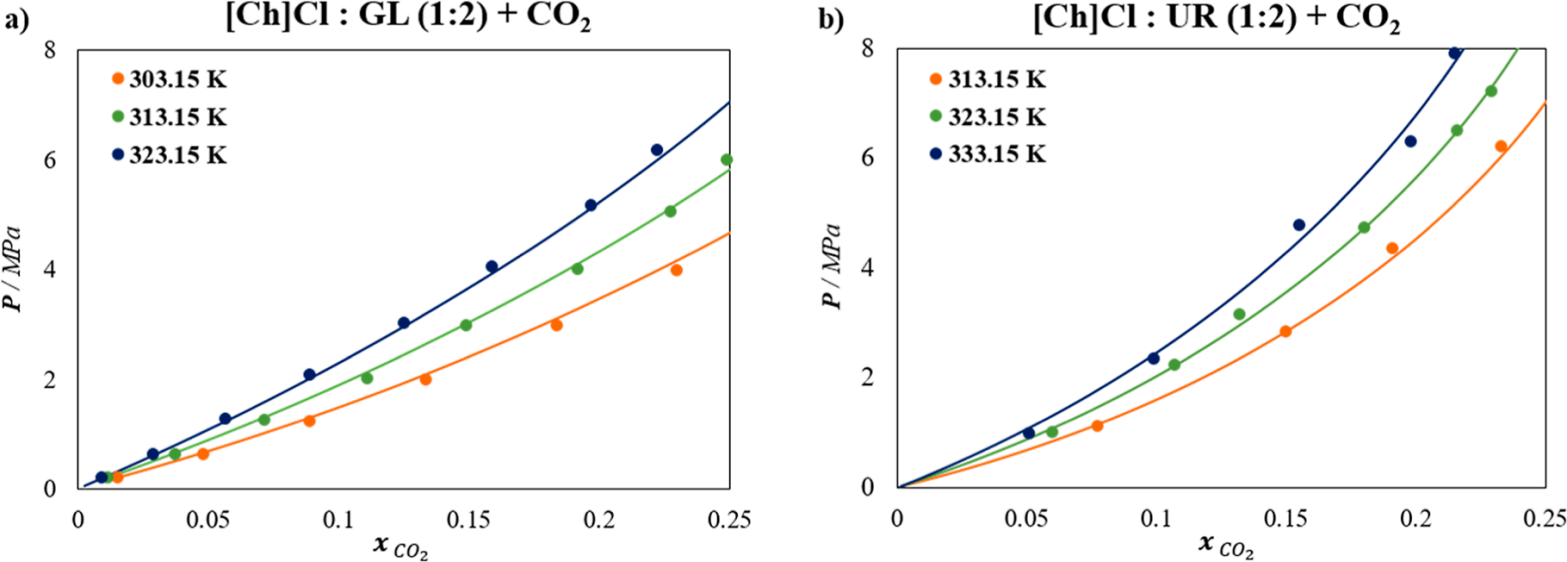
Solubility of CO_2_ at different temperatures in (a) [Ch]­Cl:GL (1:2) DES, and (b) [Ch]­Cl:UR (1:2) DES. Symbols represent experimental data,^55,56^ and lines are soft-SAFT calculations.

### 3.2 Solubility of NH_3_ in [Ch]Cl Based-DESs

In a similar approach to that used for CO_2_ solubility, the solubility of NH_3_ in DESs must first be characterized by accurately describing the binary interactions. First, EG-NH_3_ has been evaluated with soft-SAFT and fitted to available experimental data.^57^ While CO_2_ was modeled as a nonassociating (but polar) compound, the associating nature of ammonia, represented by 4 sites in soft-SAFT, allows the optimization of the cross-association interactions between NH_3_ and the DESs components, given their substantial influence on the thermophysical property predictions in such mixtures.^52^ Notice that these interactions correspond to the e–A (negative–positive) and H–B (positive–negative) interactions between NH_3_ and EG, while e–B and H–A were set to zero (same sign). To optimize these interactions, suitable values for cross-association parameters were determined within a logical range based on the physical interpretation of chemical reactions. Specifically, the correction factor for the cross-association energy, $\alpha_{EG-{NH}_{3}}^{HB}=1$ .386, was found from fitting to experimental VLE data for the EG + NH_3_ system (Figure 3a), as the default value ( $\alpha_{EG-{NH}_{3}}^{HB}=1$ ) did not satisfactorily describe the experimental behavior. This adjustment increased the cross-association energy between EG and NH_3_ from 2231.64 to 3093 K, reinforcing the presence of strong hydrogen bonding interactions, as also reported through Ab Initio Molecular Dynamics evidences by Malik and Kashyap.^58^

**Figure 3:**
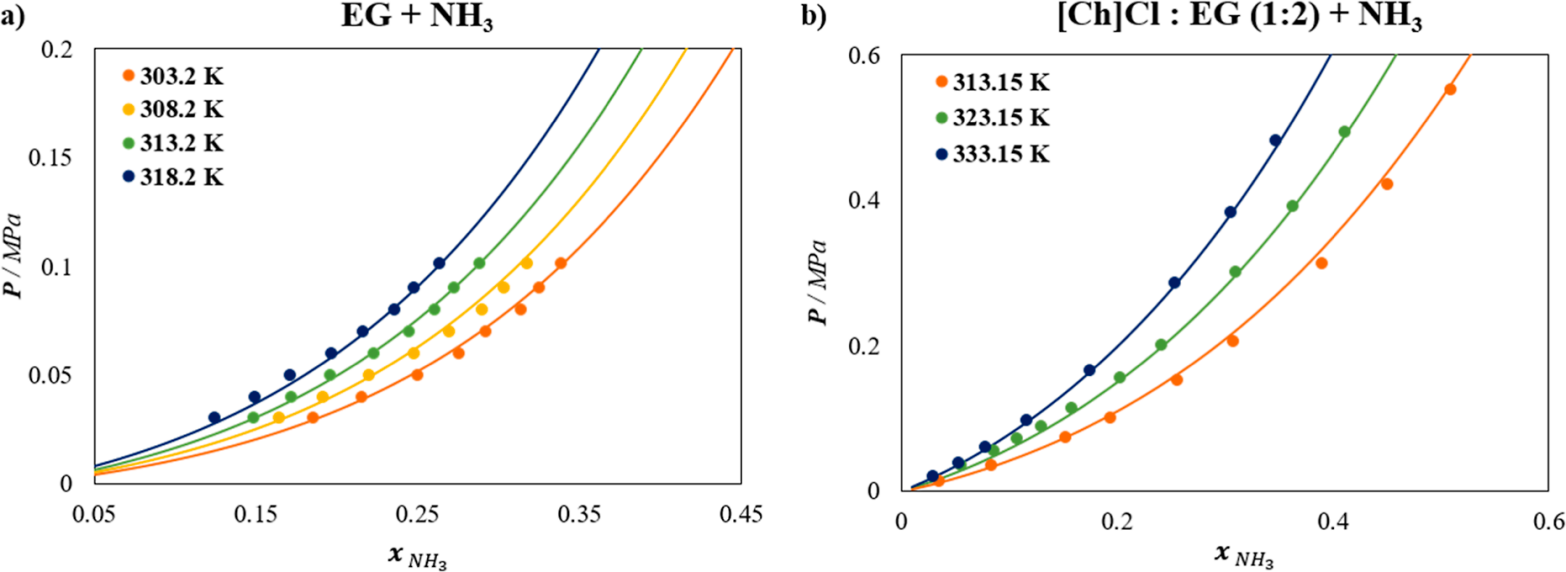
Solubility of NH_3_ at different temperatures in (a) EG, and (b) [Ch]­Cl:EG (1:2) DES. Symbols represent experimental data,^57,59^ and lines are soft-SAFT calculations.

Additionally, the size binary interaction parameter $\eta_{EG-{NH}_{3}}=$ 0.9 was also fitted to better capture the behavior of the system in the whole range of compositions, while the dispersive energy binary parameter ξ_EG–NH_3__ was set to 1, as the complex interactions are assumed to be effectively captured by the cross-association value. These fittings were performed at an intermediate temperature of 313.2 K and then applied in a transferable manner to predict the solubility at other temperatures. The results of the soft-SAFT description of the VLE for the EG–NH_3_ mixture at various temperatures are presented in Figure 3a. Good agreement between experimental data^57^ and the soft-SAFT model is found, with an % AAD of 2.01%.

Once the EG–NH_3_ mixture has been accurately described, the ternary system [Ch]­Cl:EG + NH_3_ is evaluated and taken as the benchmark system to estimate all necessary binary parameters. As far as the EG–NH_3_ interactions are transferred from the previous study (Figure 3a), and no binary parameters are required for the HBA/HBD, the remaining interaction parameters between the salt ([Ch]­Cl) and NH_3_ were fitted to adjust the ternary mixture of [Ch]­Cl:EG (1:2) + NH_3_, obtaining $\xi_{\left[Ch\right]Cl-{NH}_{3}}$ = 0.80 and $\eta_{\left[Ch\right]Cl-{NH}_{3}}$ = 1.030 across all isotherms. Figure 3b shows the results for [Ch]­Cl:EG (1:2) + NH_3_, demonstrating an excellent description of the NH_3_ solubility across various isotherms for this DES, with an % AAD of 4.45%.

The ξ and η parameters between NH_3_ and [Ch]­Cl were subsequently transferred to mixtures of NH_3_ with the other two [Ch]­Cl-based DESs studied, GL and UR. No further adjustments for $\xi_{HBD-{NH}_{3}}$ were necessary, while the remaining $\eta_{HBD-{NH}_{3}}$ values were optimized to a constant value of 0.97 for GL and UR using the intermediate temperature isotherm, with predictions extended to the other two isotherms. Finally, cross-association energy parameters were also determined between NH_3_ and the other HBDs, requiring the use of correction factors $\alpha_{GL-{NH}_{3}}^{HB}=1.442$ and $\alpha_{UR-{NH}_{3}}^{HB}=1.359$ , respectively. These adjustments increased the cross-association energy between GL and NH_3_ from 2370.13 to 3418 K, and between UR and NH_3_ from 1960.67 to 2665 K, indicating strong hydrogen bonding interactions. These parameters were also fitted using an intermediate isotherm at 323.15 K. A summary of all the necessary η_*ij*_ and the correction factor for the cross-association energy values for quantitatively describing the NH_3_ solubility in DESs is provided in Table S3 of the Supporting Information.

The description of the solubility of NH_3_ in [Ch]­Cl-based systems with GL and UR in a (1:2) ratio is illustrated in Figure 4, with % AAD values of 6.94% and 3.01%, respectively. It is important to remark that no degeneracy of the model is observed when predicting the behavior of other isotherms not used in the fitting.

**Figure 4:**
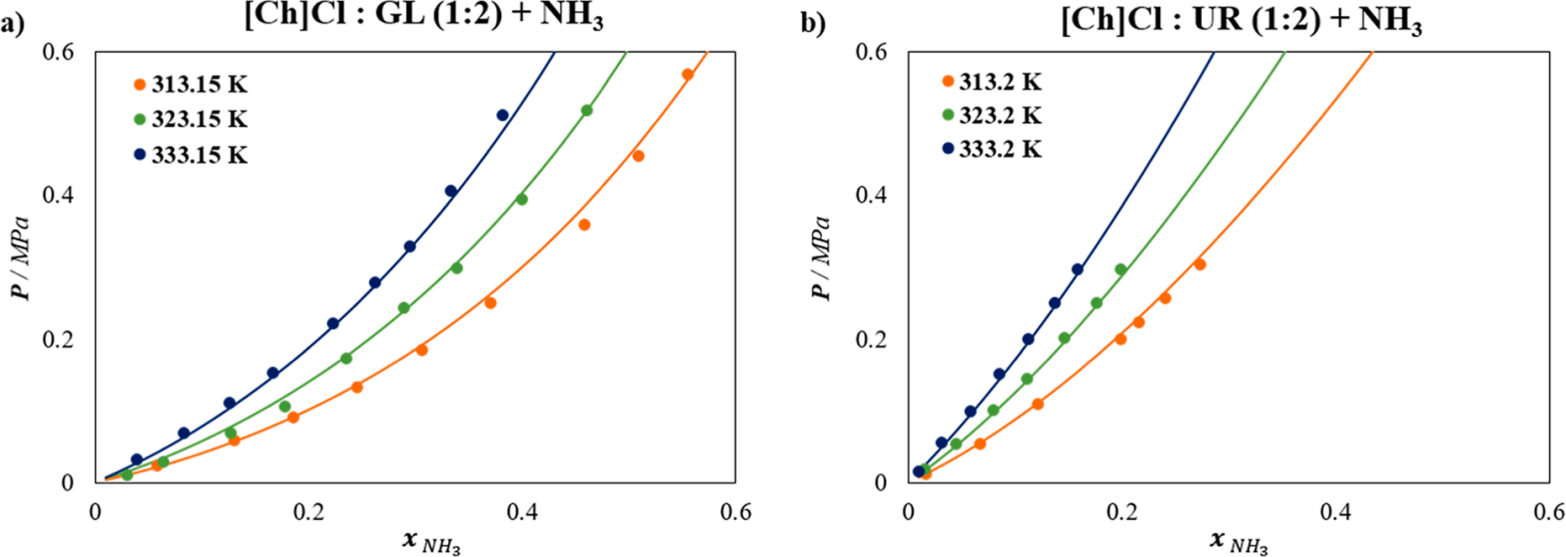
Solubility of NH_3_ at different temperatures in (a) [Ch]­Cl:GL (1:2) DES, and (b) [Ch]­Cl:UR (1:2) DES. Symbols represent experimental data,^24,59^ and lines are soft-SAFT calculations.

### 3.3 Enthalpy and Entropy of Dissolution

Beyond solubility, the absorption enthalpy and entropy also play key roles in CO_2_ and NH_3_ uptake by DESs. These properties offer valuable insights into the energetic and structural changes occurring upon gas dissolution in DESs,^60^ and can be predicted using soft-SAFT and standard thermodynamic expressions

6 $${\Delta H}_{dis,i}=-RT^{2}{\left(\frac{d\ln P_{i}}{dT}\right)}_{x_{i}}$$

7 $${\Delta S}_{dis,i}=-RT{\left(\frac{d\ln P_{i}}{dT}\right)}_{x_{i}}$$

where Δ*H*_dis,*i*_ and Δ*S*_dis,*i*_ are the molar enthalpy and entropy of dissolution, *R* is the universal gas constant (8.3145 J·mol^–1^·K^–1^), *P*_*i*_ is the partial pressure of the dissolved of gas *i*, *T* is the overall system temperature, and *x*_*i*_ is the mole fraction of the dissolved gas *i* in the DESs (fixed at 0.01 to represent dilute conditions). The corresponding values for each gas in the respective DES are summarized in Table S4 of the Supporting Information.

The results show that Δ*H*_dis_ values are consistently more negative for NH_3_ than for CO_2_ across all DESs, indicating that NH_3_ dissolution is more exothermic. Similarly, Δ*S*_dis_ values are negative for both gases, mainly attributed to gas condensing, being also more negative for NH_3_. The reduction in entropy due to this phase transition is not compensated by the entropy generated from the disruption of CO_2_ or NH_3_ into the ordered structure of the DESs.

### 3.4 Impact of the HBD and HBA/HBD Proportion in the Ideal Selectivity

The capability of an absorbent to selectively capture a specific gas from a mixture is essential for optimizing gas recycling and separation processes. In this regard, evaluating the selectivity between CO_2_ and NH_3_ appears as a key performance indicator to check the potential of DESs for this separation. Consequently, the ideal selectivity of CO_2_ and NH_3_ in the three DESs was calculated to prescreen their efficiency in separating these gases. The ideal selectivity at infinite dilution is obtained as the ratio of the effective Henry’s law constants for the two pure gases. It indicates the relative solubility of each gas in the DES at a given isotherm,^61^ as follows

8 $${\beta_{i/j}}^{T}=\frac{{k_{H,eff}^{j}}^{T}}{{k_{H,eff}^{i}}^{T}}=\frac{\lim_{x_{j}\to 0}{\left(\frac{P}{x_{j}}\right)}^{T}}{\lim_{x_{i}\to 0}{\left(\frac{P}{x_{i}}\right)}^{T}}$$

where *k*_H,eff_^*i*^ represents the effective Henry’s law constant^62^ for gas *i* at temperature *T*, *x*_*i*_ denotes the molar fraction of compound *i* in the liquid, and β_*i*/*j*_ represents the ideal selectivity at infinite dilution of the compound *j* concerning the compound *i*.

In this work, soft-SAFT is used to predict pressure data as solubility approaches zero for each isotherm and compound, allowing to evaluate the effective Henry’s law constants and the ideal selectivity. The results are graphically shown in Figure 5a at 313.15 K, while the list of values at all temperatures investigated are included in Tables S5 and S6 of the Supporting Information. The effective Henry’s constants are directly linked to the solubility measurements shown in Figures 1–4. In general, all DESs exhibit larger values of effective Henry’s constants (i.e., lower affinity) for CO_2_ than for NH_3_. The values of CO_2_ absorption are in agreement with those reported in the literature for ammonium salt-based DESs.^35,36^ As illustrated in Figure 5a (blue bars graph, left axis), [Ch]­Cl:UR (1:2) shows the lowest *k*_H,eff_ for CO_2_, indicating a higher absorption capacity, followed by [Ch]­Cl:GL (1:2) and [Ch]­Cl:EG (1:2). Contrarily, [Ch]­Cl:UR shows the highest *k*_H,eff_ for NH_3_ (orange bars graph, left axis), indicating a lower absorption capacity, while the other DESs exhibit lower similar values of *k*_H,eff_ for NH_3_.

**Figure 5:**
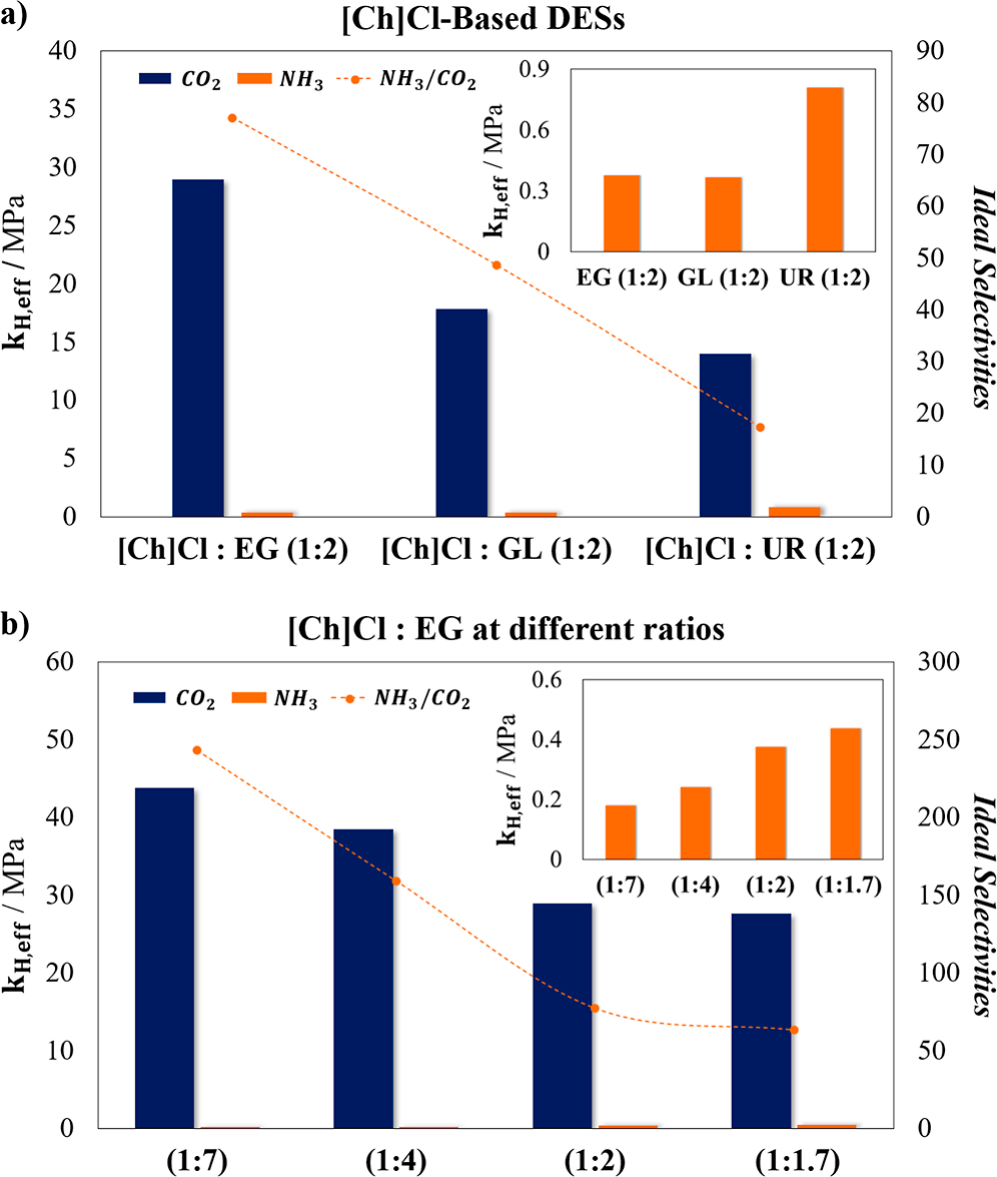
Calculated effective Henry’s constants (*k*_H,eff_) for CO_2_ and NH_3_ (bars graph, left axis) and ideal selectivity ( $\beta_{{NH}_{3}/{CO}_{2}}$ , symbols and lines, right axis) at 313.15 K: (a) for [Ch]­Cl:EG (1:2), [Ch]­Cl:GL (1:2), and [Ch]­Cl:UR (1:2); and (b) for [Ch]­Cl:EG at different molar ratios.

Regarding the ideal selectivity of NH_3_/CO_2_ shown in Figure 5a (symbols and lines, right axis), the highest $\beta_{{NH}_{3}/{CO}_{2}}$ value (77.108) among the investigated DESs for the binary mixtures is achieved with [Ch]­Cl:EG (1:2), indicating the best selectivity and preference for NH_3_ in this mixture. Based on these preliminary results, [Ch]­Cl:EG is preselected as the most suitable DES for NH_3_–CO_2_ separation.

While the [Ch]­Cl:EG HBA:HBD combination seems to offer the most promising performance, it is interesting to check the influence of the molar ratio of this DES on the selectivity of NH_3_ over CO_2_. Of course, this range will be limited by the eutectic point, so it is known that a minimum ratio of approximately 1:1.7 is needed to avoid the solidification of the solvent in the range of temperatures studied.^63,64^

Using soft-SAFT, the solubility of both gases in [Ch]­Cl:EG is predicted at different HBA:HBD proportions and compared to the [Ch]­Cl:EG (1:2) system. The analysis includes higher ratios (1:4) and (1:7) of EG, for which experimental data were available.^65^ Even in the absence of data, the low ratio (1:1.7) was also predicted to assess the effect of reduced EG content. As shown in Figure S3 of the Supporting Information, the soft-SAFT model provides good agreement with the available NH_3_ experimental data, yielding an overall % AAD of 8.07%. All calculations were made in a fully predictive manner using the molecular parameters optimized for the [Ch]­Cl:EG (1:2) system (Figure 3), without the need for any additional parameter adjustments, demonstrating the robustness and transferability of the model to different DES compositions. In the case of CO_2_, no experimental solubility data were available for [Ch]­Cl/:EG at different ratios, and soft-SAFT predictions at various molar ratios are presented in Figure S4 of the Supporting Information.

The results of these absorption-isotherms predictions *k*_H,eff_^*i*^ of CO_2_ and NH_3_, as well as $\beta_{{NH}_{3}/{CO}_{2}}$ , were estimated for the four DESs. Figure 5b illustrates both, *k*_H,eff_^*i*^ and $\beta_{{NH}_{3}/{CO}_{2}}$ , for [Ch]­Cl:EG at different molar ratios, while the exact values are summarized in Table S7 of the Supporting Information.

The results reveal that increasing the molar ratio of [Ch]­Cl:EG from 1:2 to 1:7 decreases the CO_2_ absorption capacity, as evidenced by the increase in *k*_H,eff_^*i*^ from 28.985 to 43.77. Conversely, this change increases the NH_3_ absorption capacity, indicated by the decrease in the corresponding *k*_H,eff_^*i*^ from 0.3759 to 0.18. When the molar ratio is decreased from 1:2 to 1:1.7, the opposite effect occurs. As a result, increasing the molar ratio from 1:2 to 1:7 leads to an approximate 215% increase in the ideal selectivity factor $\beta_{{NH}_{3}/{CO}_{2}}$ , rising from 77.108 to 243.167. These findings suggest that tuning the HBA/HBD molar ratio is a promising strategy for enhancing NH_3_ over CO_2_ selectivity in [Ch]­Cl:EG-based DESs.

### 3.5 Separation Performance in an NH_3_/CO_2_ Tail Gas from Melamine Production

The ideal selectivity calculated from effective Henry’s coefficients is an approximation based on the infinite dilution behavior of pure compounds (CO_2_ and NH_3_ in this study). This method does not account for the competition between gases, providing only a preliminary indication of each DES’s separation capacity. To accurately assess gas separation efficiency, competitive selectivity is evaluated here to simulate the recovery of NH_3_ from melamine production tail gas. According to Duan et al.,^8^ a melamine tail gas produced in China contains 7.6% N_2_, 0.4% H_2_O, 55% NH_3_, and 37% CO_2_ in mole fraction. In our study, this residual mixture has been simplified to a binary system containing 60% NH_3_ and 40% CO_2_ in mole fraction. Although a detailed analysis should include all the components present in the tail gas, our study focuses on the main constituents to enable an initial screening of the most suitable solvent. Indeed, it is expected that N_2_ exhibit minimal solubility, acting mostly as an inert.^8^ Concerning water, its content is very low, and its main effect in the system is expected to be a reduction of DES viscosity rather than having a significant influence on the relative solubilities of CO_2_ and NH_3_ in the solvent.

In this case, the values of competitive selectivity (*S*) calculated for these gas mixtures are obtained through eq 9

9 $$S_{i/j}=\frac{y_{j}/x_{j}}{y_{i}/x_{i}}$$

where *x* and *y* are the liquid and vapor molar fractions of each gas in the mixture, respectively. These compositions can be obtained from liquid–vapor flash calculations using soft-SAFT, which are computed by an iterative procedure that solves a modified multiphase Rachford–Rice equation mass balance.^66,67^ The calculation assumes an initial global composition with a DES mole fraction of 0.7 (*z*_DES_ = 0.7) and 0.3 for the melamine tail gas blend ( $z_{{NH}_{3}+{CO}_{2}}=0.3$ ), the latter on a 60:40 mol % basis, as previously specified. A schematic diagram of the separation process is shown in Figure 6a. The separation performance of NH_3_/CO_2_ blends at 313.15 K and different pressures is shown in Figure 6b,c.

**Figure 6:**
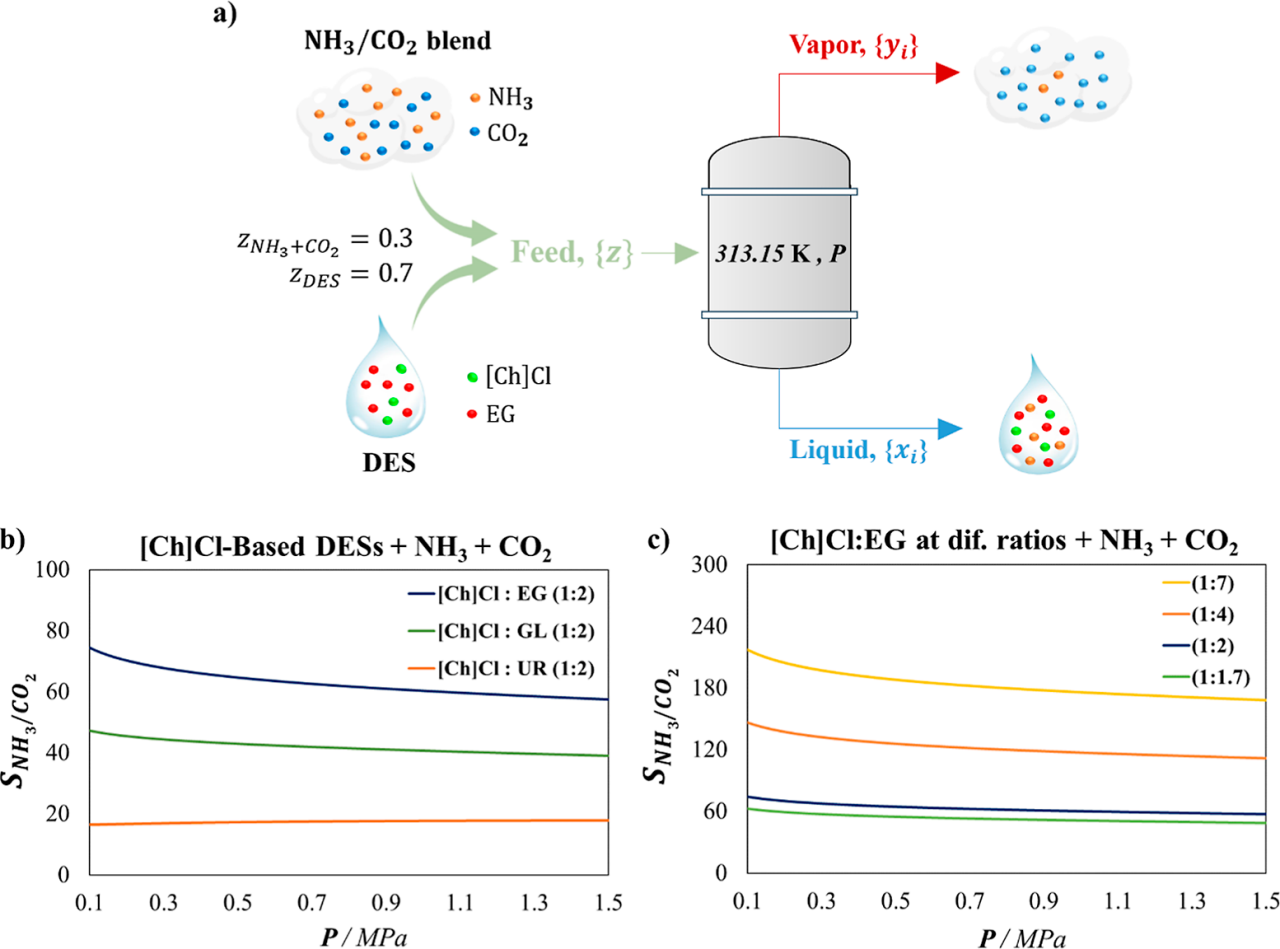
(a) Illustration of vapor–liquid flash calculations via soft-SAFT EoS depicting the separation performance of NH_3_/CO_2_ blends at 313.15 K and different pressures. NH_3_ competitive selectivity over CO_2_ in (b) [Ch]­Cl-based DESs, and (c) [Ch]­Cl:EG at different proportions. Global molar composition of the analyzed mixture of *z*_DES_ = 0.7 and $z_{{CO}_{2}+{NH}_{3}}=0.3$ modeled using soft-SAFT EoS. The CO_2_ + NH_3_ blend contains 60% NH_3_ and 40% CO_2_ in mole fraction.

Overall, significant potential for achieving a high separation efficiency in the NH_3_–CO_2_ separation at the specified conditions is observed. The predicted competitive selectivity closely aligns with the ideal selectivity at infinite dilution, as seen when comparing Figure 5 with Figure 6. This correlation is especially evident at low pressures, where ideal conditions are predominant. However, while low pressures enhance separation performance, they come at the expense of reduced sorption capacity, as indicated in Figures 1–4, leading to a lower NH_3_ recovery in quantitative terms. Conversely, high-pressure conditions do not yield favorable outcomes, as they decrease the NH_3_–CO_2_ separation efficiency for [Ch]­Cl-based DESs. Therefore, based on this analysis, operating under moderate pressure conditions is recommended for efficient NH_3_–CO_2_ separation. Among the studied DESs, [Ch]­Cl:EG (1:7) stands out as the best choice for NH_3_ capture from melamine tail gas streams.

## 4 Conclusions

Aimed to find an optimal recovery process for the separation of CO_2_ and NH_3_ in melamine tail gas streams, a comprehensive study was conducted to model the solubility of these gases in three [Ch]­Cl-based DESs, with EG, UR, and GL as HBDs, using the soft-SAFT EoS. The soft-SAFT calculations effectively described the isotherms of CO_2_ and NH_3_ in the three studied DESs over the temperature range of 303.15 to 333.15 K, showing excellent agreement with experimental data. Temperature-independent binary parameters for energy and size were adjusted between the DESs components and the studied gases to achieve this level of accuracy. Later, the soft-SAFT EoS was used to predict essential properties, including enthalpy and entropy of dissolution, effective Henry’s constants, and ideal selectivity. Among the investigated DESs, [Ch]­Cl:UR (1:2) demonstrated the highest CO_2_ solubility, whereas [Ch]­Cl:EG (1:2) exhibited the highest NH_3_ absorption capacity at 313.15 K. The competitive selectivity of the NH_3_/CO_2_ mixture (60/40 mol % basis, simulating the tail gas composition from melamine production) in the DESs was evaluated at 313.15 K and in the 0.1–1.5 MPa pressure range. For the separation of NH_3_ from CO_2_ mixtures in melamine tail gas streams at 313.15 K, [Ch]­Cl:EG (1:7) at moderate pressures emerged as the most effective DES among those studied solvents and conditions. Following this computational approach, experiments can be conducted with the selected entrainer to validate the results and refine the optimal conditions for the separation process, providing a more environmentally friendly solution to current industrial options.

## Supplementary Material



## Notes

Molecular structure representations; VLE curves and parameter comparison for NH_3_; soft-SAFT solubility predictions and flash separation performance for CO_2_ and NH_3_ in [Ch]­Cl-based DESs at different HBA/HBD ratios; calculated enthalpy and entropy of absorption; effective Henry’s law constants and NH_3_/CO_2_ ideal selectivities values at various temperatures (PDF)
