# Supplementary material for: Finding Sustainable Deep Eutectic Solvents for an Efficient Separation of CO2 and NH3 in Melamine Production with Soft-SAFT
Source: ACS Sustain Chem Eng. 2025 Sep 4;13(36):15097–106. doi: 10.1021/acssuschemeng.5c06167 (PMC12442516; doi:10.1021/acssuschemeng.5c06167)
Supplement: Supplementary file 1 [file sc5c06167_si_001.pdf]

Supporting Information

Finding Sustainable Deep Eutectic Solvents for an Efficient Separation of CO_2 and NH_3 in Melamine Production with soft-SAFT

Luan Vittor Tavares Duarte de Alencar[a,b], Sabrina Belén Rodríguez-Reartes[a,c,d], Frederico Wanderley Tavares[b,e], Fèlix Llovell[a*]

[a]Department of Chemical Engineering, ETSEQ, Universitat Rovira i Virgili, Avinguda Països Catalans 26, 43007, Tarragona, Spain.

[b]Programa de Engenharia Química (PEQ/COPPE), Universidade Federal do Rio de Janeiro (UFRJ), Athos da Silveira Ramos Avenue, 149 - Block G -Ilha do Fundão, 21941-909, Rio de Janeiro, RJ, Brazil.

[c]Departamento de Ingeniería Química, Universidad Nacional del Sur (UNS), Avda. Alem 1253, Bahía Blanca, (8000), Argentina.

[d]Planta Piloto de Ingeniería Química – PLAPIQUI (UNS-CONICET), Camino "La Carrindanga" Km 7, Bahía Blanca, (8000), Argentina.

[e]Engenharia de Processos Químicos e Bioquímicos, Escola de Química (EPQB), Universidade Federal do Rio de Janeiro (UFRJ), Athos da Silveira Ramos Avenue, 149 - Block E - Ilha do Fundão, 21941-909, Rio de Janeiro, RJ, Brazil.

This document contains the supplementary material of the article entitled "*Finding Sustainable Deep Eutectic Solvents for an Efficient Separation of CO_2 and NH_3 in Melamine Production with soft-SAFT*".

Number of figures: 4

Number of tables: 7

 Corresponding author.

E-mail address: felix.llovell@urv.cat

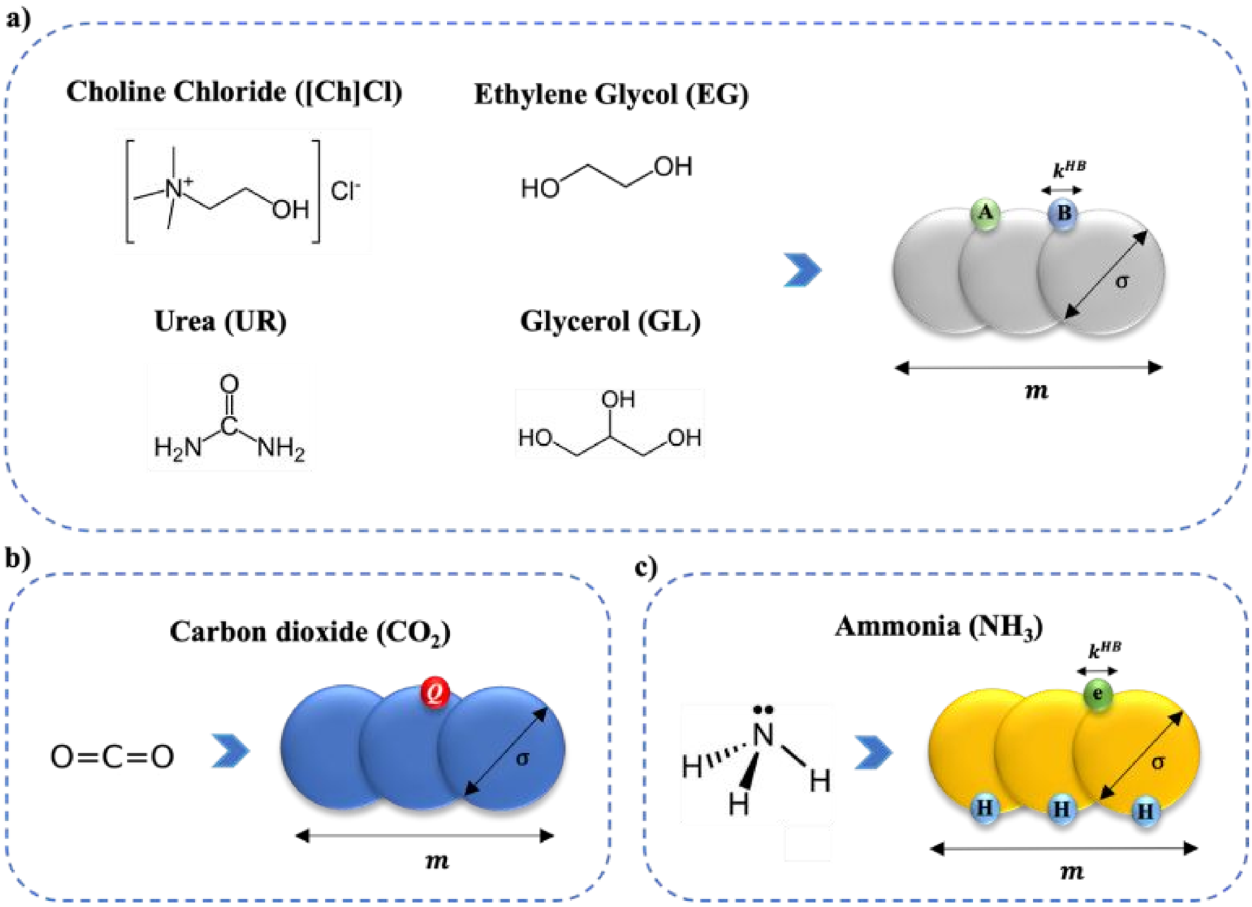

Figure S1. *Image of the chemical structure for (a) the compounds that form the DESs (b) carbon dioxide (CO_2) and (c) ammonia (NH_3), and sketches of the molecular model used to describe each compound within the soft-SAFT approach.*

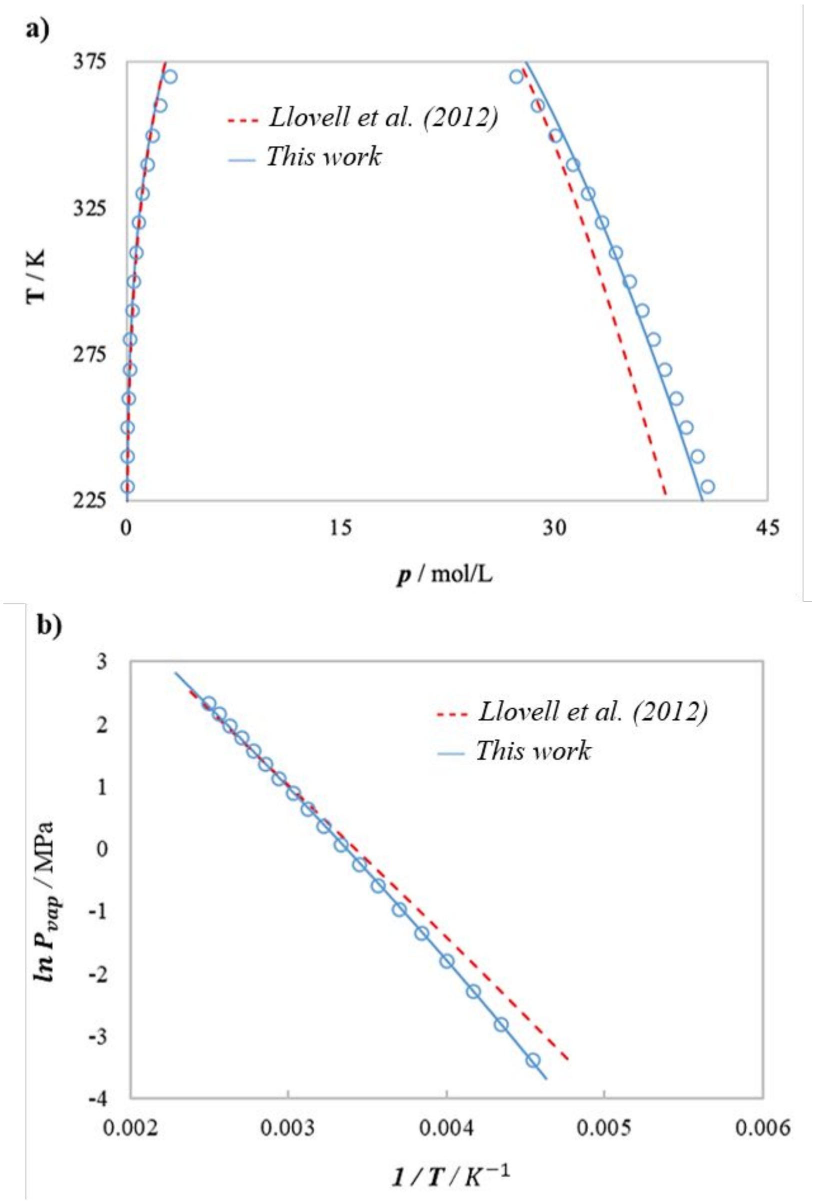

Figure S2. *Vapor-liquid equilibrium properties of ammonia: a) Phase equilibrium diagram and b) Vapor pressure in the Clausius−Clapeyron representation. The experimental data (circles) are taken from NIST* [1]*. The lines represent the soft-SAFT calculations, with dashed lines from Llovell et al.* [2] *and solid lines from this work.*

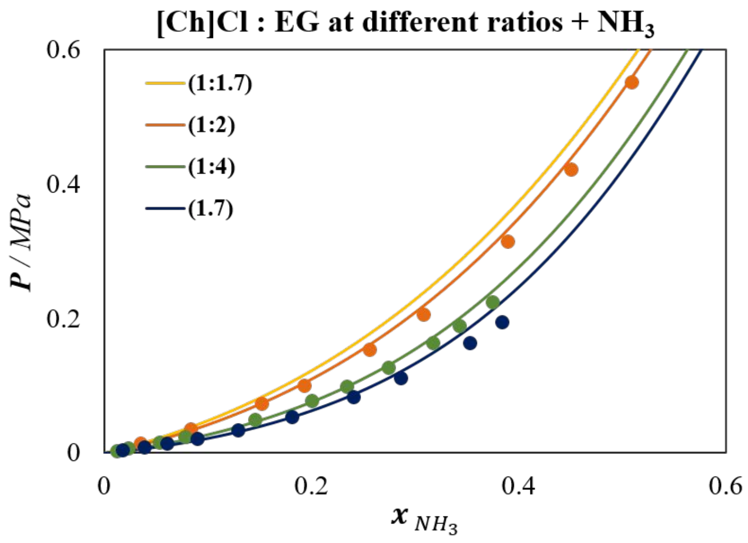

Figure S3. *Solubility of NH_3 in the DESs [Ch]Cl:EG at different HBA:HBD ratios at 313.15 K. Symbols represent experimental data* [3, 4]*, and lines are soft-SAFT calculations.*

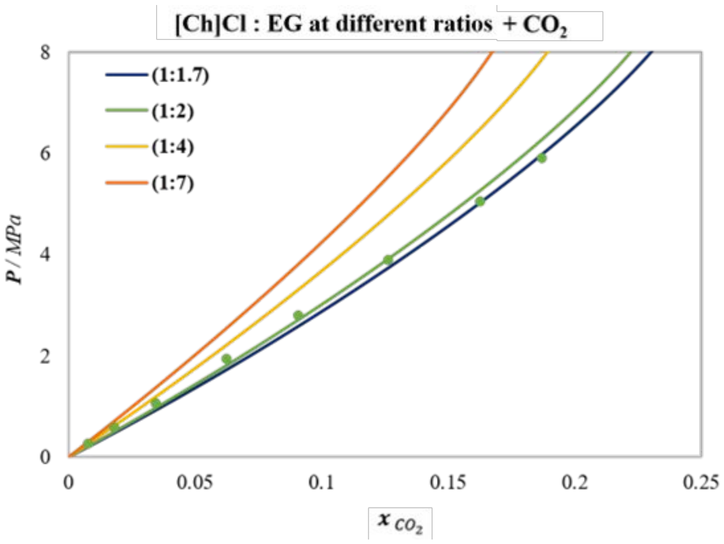

Figure S4. *Solubility of CO_2 in the DESs [Ch]Cl:EG at different HBA:HBD ratios at 313.15 K. Symbols represent experimental data* [4] *and lines soft-SAFT predictions.*

Table S1. *soft-SAFT molecular parameters optimized for NH_3 in the temperature range of 225–375 K.*

Compound	M_w (g/mol)	m	σ (Å)	ε/k_B (K)	ε^{HB}/k_B (K)	k^{HB} (Å^3)	Reference
NH_3	17.04	1.418	2.974	280.5	483.0	2160.5	[2]
NH_3	17.04	1.873	2.679	236.5	1136	1498.0	This work

.

Table S2. *soft-SAFT energy binary interaction parameters (ξ_{ij}) adjusted in this work for interactions with CO_2.*

Compound i	Compound j	ξ_{ij}
[Ch]Cl	CO_2	1.100
EG	CO_2	0.886
GL	CO_2	1.018
UR	CO_2	1.095

Table S3. *soft-SAFT size binary interaction parameters (η_{ij}) and correction factor for the cross-association energy (α_{ij}^{HB}) adjusted in this work for interactions with NH_3.*

Compound i	Compound j	η_{ij}	α_{ij}^{HB}
[Ch]Cl	NH_3	1.030	1.000
EG	NH_3	0.900	1.386
GL	NH_3	0.970	1.442
UR	NH_3	0.970	1.359

Table S4. *Calculated Enthalpy and Entropy of Dissolution for CO_2 and NH_3 in DESs from 303.15 to 333.15 K at a DES Liquid Phase Molar Composition of 0.01.*

DES	ΔH_{dis} /kJ.mol^{-1}		ΔS_{dis} /J. K^{-1}.mol^{-1}	
	**CO_2**	**NH_3**	**CO_2**	**NH_3**
**[Ch]Cl : EG (1:2)**	-12.97	-29.56	-40.82	-93.06
**[Ch]Cl : GL (1:2)**	-18.34	-31.51	-57.69	-99.16
**[Ch]Cl : UR (1:2)**	-23.10	-33.74	-72.65	-106.15

Table S5. *Effective Henry's law constants determined for the absorption of CO_2 and NH_3 at different temperatures in the selected DESs (MPa).*

DES	CO_2 Absorption				NH_3 Absorption		
	303.15 K	313.15 K	323.15 K	333.15 K	313.15 K	323.15 K	333.15 K
[Ch]Cl : EG (1:2)	24.279	28.985	33.628	-	0.3759	0.5208	0.7013
[Ch]Cl : GL (1:2)	13.857	17.840	21.800	-	0.3668	0.5215	0.7128
[Ch]Cl : UR (1:2)	-	13.995	17.965	21.973	0.8101	1.1748	1.6193

Table S6. *The ideal selectivity (β) of NH3/CO2 at different temperatures in the selected DESs.*

DES	*Ideal* Selectivity (β_{NH_3/CO_2})		
	313.15 K	323.15 K	333.15 K
[Ch]Cl : EG (1:2)	77.108	64.570	-
[Ch]Cl : GL (1:2)	48.637	41.802	-
[Ch]Cl : UR (1:2)	17.276	15.292	13.569

Table S7. *Effective Henry's law constants (MPa) for CO_2 and NH_3 absorption in [Ch]Cl:EG DESs at different molar ratios, and corresponding NH_3/CO_2 ideal selectivity (β), all determined at 313.15 K.*

DES	CO_2 absorption	NH_3 absorption	β_{NH_3/CO_2}
[Ch]Cl : EG (1:7)	43.770	0.1800	243.167
[Ch]Cl : EG (1:4)	38.450	0.2417	159.082
[Ch]Cl : EG (1:2)	28.985	0.3759	77.108
[Ch]Cl : EG (1:1.17)	27.671	0.4382	63.145

References

(1) NIST. Chemistry Webbook.

(2) Llovell, F.; Marcos, R. M.; MacDowell, N.; Vega, L. F. Modeling the Absorption of Weak Electrolytes and Acid Gases with Ionic Liquids Using the Soft-SAFT Approach. *The Journal of Physical Chemistry B* **2012**, *116* (26), 7709-7718. DOI: https://doi.org/10.1021/jp303344f.

(3) Huang, J.-Y.; Jiang, W.-J.; Xiao, P.; Fan, Z.-T.; Zhong, F.-Y.; Peng, H.-L.; Du, J.; Huang, K. Physical Properties and NH3 Solubilities of Deep Eutectic Solvents Formed by Choline Chloride and Glycols. *Fluid Phase Equilibria* **2021**, *529*, 112871. DOI: https://doi.org/10.1016/j.fluid.2020.112871.

(4) Duan, X.; Gao, B.; Zhang, C.; Deng, D. Solubility and thermodynamic properties of NH3 in choline chloride-based deep eutectic solvents. *The Journal of Chemical Thermodynamics* **2019**, *133*, 79-84. DOI: https://doi.org/10.1016/j.jct.2019.01.031.
